# The Effects of Disturbance on Hypothalamus-Pituitary-Thyroid (HPT) Axis in Zebrafish Larvae after Exposure to DEHP

**DOI:** 10.1371/journal.pone.0155762

**Published:** 2016-05-25

**Authors:** Pan-Pan Jia, Yan-Bo Ma, Chun-Jiao Lu, Zakaria Mirza, Wei Zhang, Yong-Fang Jia, Wei-Guo Li, De-Sheng Pei

**Affiliations:** 1 College of Life Science, Henan Normal University, Xinxiang, 453007, China; 2 Research Center for Environment and Health, Eco-Environmental Institute for Three Gorges, Chongqing Institute of Green and Intelligent Technology, Chinese Academy of Sciences, Chongqing, 401122, China; INIA, SPAIN

## Abstract

Di-(2-ethylhexyl) phthalate (DEHP) has the potential to disrupt the thyroid endocrine system, but the underlying mechanism is unknown. In this study, zebrafish (*Danio rerio*) embryos were exposed to different concentrations of DEHP (0, 40, 100, 200, 400 μg/L) from 2 to 168 hours post fertilization (hpf). Thyroid hormones (THs) levels and transcriptional profiling of key genes related to hypothalamus-pituitary-thyroid (HPT) axis were examined. The result of whole-body thyroxine (T4) and triiodothyronine (T3) indicated that the thyroid hormone homeostasis was disrupted by DEHP in the zebrafish larvae. After exposure to DEHP, the mRNA expressions of thyroid stimulating hormone (*tshβ*) and corticotrophin releasing hormone (*crh*) genes were increased in a concentration dependent manner, respectively. The expression level of genes involved in thyroid development (*nkx2*.*1* and *pax8*) and thyroid synthesis (sodium/iodide symporter, *nis*, thyroglobulin, *tg*) were also measured. The transcripts of *nkx2*.*1* and *tg* were significantly increased after DEHP exposure, while those of *nis* and *pax8* had no significant change. Down-regulation of uridinediphosphate-glucuronosyl-transferase (*ugt1ab)* and up-regulation of thyronine deiodinase (*dio2)* might change the THs levels. In addition, the transcript of transthyretin (*ttr*) was up-regulated, while the mRNA levels of thyroid hormone receptors (*trα* and *trβ*) remained unchanged. All the results demonstrated that exposure to DEHP altered the whole-body thyroid hormones in the zebrafish larvae and changed the expression profiling of key genes related to HPT axis, proving that DEHP induced the thyroid endocrine toxicity and potentially affected the synthesis, regulation and action of thyroid hormones.

## Introduction

Phthalates acid esters (PAEs) as plasticizers are wildly used in different kinds of plastic products, such as food contact materials, blood bags, toys, clothing, adhesives, plastic coating, lubricants, cosmetics and electronics, *etc*. PAEs have been detected in air, soil and water, and caused serious problems to the wild animals and human beings. Among the PAEs family, di-(2-ethylhexyl) phthalate (DEHP) is the most extensively used PAEs, accounting for approximately 50% of total plasticizer production [1, 2]. Due to wide usage and constant release into the environment, DEHP exists in rivers, lakes and running water at different concentration levels [3, 4]. In Germany, the concentration of DEHP in water systems was found as high as 98 μg/L, even higher to 247 μg/L in the drinking water in Croatia [5–7]. In China, previous studies reported that the concentrations of DEHP in surface waters ranged from 5 μg/L to 76 μg/L, and in Wuhan section of the Yangtze River from 3.9 μg/L to 55 μg/L [8–10]. Studies showed that DEHP released into environment can cause severe health risks [11, 12], but its developmental risks on water system organisms are still elusive.

Phthalate esters can cause endocrine disruptions in organisms by interfering with reproduction and the thyroid hormone (TH) systems [13–15]. Previous studies proved that DEHP can impair reproduction system by affecting critical factors in oogenesis and spermatogenesis in zebrafish, and disrupt the endocrine of sex hormones in Chinese rare minnow [16–18]. Moreover, DEHP could cause a risk to human health by disrupting reproductive system [19, 20]. Recent studies showed that DEHP, as an endocrine disruptor, can disturb the thyroid hormone homeostasis. Chen *et al*. proved that DEHP/Mono-(2-ethylhexyl) phthalate (MEHP) disturbed the body’s hormone stability and posed a serious threat to animal development [21]. Wang *et al*. reported that the accumulation of DEHP caused endocrine disruption in medaka embryos [22]. Zhai *et al*. showed that MEHP, the hydrolytic metabolite of the DEHP, induced the thyroid endocrine disruption during zebrafish embryos development [23]. However, as one of the most used plasticizers that directly released into the environment, especially into the water systems, the toxicity of DEHP to aquatic organism was seldom reported and should arise our high attention.

Thyroid hormones (THs) play important roles in the processes of differentiation, growth, metabolism, development, growth, and reproduction physiologic actions in fish [24]. The thyroid endocrine system is controlled by hypothalamus–pituitary–thyroid axis (HPT), which is responsible for the thyroid hormone synthesis, secretion, transport and metabolism [25]. In mammals, the hypothalamus secretes the thyrotropin-releasing hormone (TRH), which stimulates the secretion of thyroid-stimulating hormone (TSH) and regulates THs synthesis in HPT axis. While in amphibians and teleosts, the secretion of TSH is stimulated by corticotrophin-releasing hormone (CRH) [26]. In teleosts, THs in plasma can bind to THs transport transthyretin (TTR), and only the free ones enter into target cells to launch a response [27]. Endocrine disrupting chemicals (EDCs) can disrupt the thyroid hormone stability and HPT axis in fish by altering hormone levels, enzyme activities and the expression of genes. Those alterations can be credibly used to evaluate the chemicals effect on the thyroid endocrine disruption [28, 29]. However, the underlying mechanisms of DEHP toxicity on thyroid endocrine system in fish is still not completely clarified.

Zebrafish are widely used in the toxicological researches because of its small size, easy culture, high reproductive performance, rapid organogenesis and sensitivity to the harmful chemicals [30]. Moreover, the thyroid system in zebrafish is similar to mammalian, which can provide a valuable reference for human beings [31]. Therefore, in the present study, zebrafish were used to reveal the mechanisms of the HPT axis disturbance after exposure to DEHP.

## Methods and Materials

### Ethics statement

The animal protocol for this study was approved by the Animal Care and Use Committee of Chongqing in China and by the Institutional Animal Care and Use Committee of Chongqing Institute of Green and Intelligent Technology, Chinese Academy of Sciences (Approval ID: ZKCQY0063).

### Chemicals

DEHP (No: ALR-097N, 99.6%) was purchased from AccuStandard (New Haven, CT, USA) and dissolved in dimethyl sulfoxide (DMSO, CAS: 67-68-5, purity≥99.5%, Sigma-Aldrich, USA). Then the DEHP stock solution A (8×10^7^ μg/L) and B (8×10^6^ μg/L) was stored at 4°C. 3-Aminobenzoic acid ethyl ester or methane-sulfonate salt (MS-222, CAS: 886-86-2, 98%) was obtained from Aladdin (Los Angeles, California, USA), which was used as an anesthetic to treat zebrafish larvae before sampling. All other chemicals used in the present study were of the analytical grade.

### Zebrafish maintenance and embryos exposure to DEHP

Wild-type strains of zebrafish (AB) were maintained in a flow-through system at a constant temperature (28°C) and 14:10 (light: dark) photoperiod. Zebrafish were fed three times every day with the newly hatched brine shrimp (*Artemia nauplii*). Normal adult zebrafish were placed in the breeding box with a ratio of 2:1 (male: female) overnight. Spawning was triggered under light and was completed within 30 min. Then embryos were collected and examined under the stereomicroscope at 2 hours post fertilization (hpf). Embryos developing normally at blastula stage were selected for the subsequent experiments. Two hundred and fifty embryos were randomly distributed into each glass beakers containing 50 mL of DEHP exposure solution (0, 40, 100, 200 and 400 μg/L). There were three replicates for each group, and the exposure concentrations were previously confirmed by a dose-ranging study based on the environmental pollutant concentration. During the experimental process, the zebrafish embryos and larvae were maintained in a biochemical incubator with 28°C and 14:10 (light: dark) condition, and the exposure solutions were renewed daily. The details for preparation of DEHP buffers was shown in S1 Table. After exposure to different concentrations of DEHP until 168 hpf, the larvae were washed with UltraPure water and anesthetized in 50 mg/L of MS-222 for random sampling. Then, the samples were immediately frozen into liquid nitrogen, and stored at -80°C for the subsequent analysis of gene expression and TH assay.

### Developmental toxicity

During DEHP exposure, the hatching rate of embryos at 72 hpf was calculated. The larvae developmental parameters for body weight, body length, survival rate and malformation rate at 168 hpf were also recorded. The body length of larvae was measured along the head-to-tail axis under a stereomicroscope.

### Total RNA isolation and quantitative RT-PCR analysis

Total RNA were extracted from 30 zebrafish larvae by using RNAiso Plus reagent (Takara Bio Inc., Shiga, Japan) according to the manufacturer’s instructions. Then, the purity and quality of the total RNA were measured by using a spectrophotometer and 1% agarose-formaldehyde gel electrophoresis with ethidium bromide staining. The synthesis of cDNA was performed with 1 μg total RNA for each sample by using the Primer Script RT Reagent Kit (Takara Bio, Shiga, Japan) according to the manufacturer’s instructions. Quantitative real-time PCR (qRT-PCR) was performed by using the SYBR Green PCR Kit (Toyobo, Tokyo, Japan) on the ABI 7300 System (PerkinElmer Applied Biosystems, Foster City, CA, USA). The primers of the selected genes were designed by using the online Primer 3 program (http://frodo.wi.mit.edu/) and their sequences were listed in Table 1. The PCR reaction was comprised of an initial denaturation step at 95°C for 3 min, followed by 40 cycles at 95°C for 20 s, 58°C for 20 s, 72°C for 45 s. The dissociation curve used to check the specificity of PCR production was acquired by adding a step of 95°C for 15 s, 60°C for 1 min, 95°C for 15 s at the end of the amplification phase. Ribosomal protein L8 (*rpl8*) was chosen as the internal control according to the stability result of five candidate reference genes (S1 Fig). The expression levels of target genes were normalized to *rpl8* by using 2^−ΔΔCT^ method.

**Table 1:** Primers used for qRT-PCR in this study.

Gene name	GenBank accession No.	Primer sequence (5’-3’)	Product length (bp)
*rpl8*	NM 200713.1	F: TTGTTGGTGTTGTTGCTGGT; R: GGATGCTCAACAGGGTTCAT	116
*crh*	NM 001007379.1	F: TTCGGGAAGTAACCACAAGC; R: CTGCACTCTATTCGCCTTCC	141
*tshβ*	NM 181494.2	F: GCAGATCCTCACTTCACCTACC; R: GCACAGGTTTGGAGCATCTCA	100
*nts*	NM 001089391.1	F: GGTGGCATGAAGGCTGTAAT; R: GATACGGCATCCATTGTTGG	124
*tg*	XM 689200.5	F: CCAGCCGAAAGGATAGAGTTG; R: ATGCTGCCGTGGAATAGGA	157
*nkx2*.*1*	NM 131589.1	F: AGGACGGTAAACCGTGTCAG; R: CACCATGCTGCTCGTGTACT	168
*pax8*	AF 072549.1	F: GAAGATCGCGGAGTACAAGC; R: CTGCACTTTAGTGCGGATGA	122
*dio1*	AY 221259.1	F: GTTCAAACAGCTTGTCAAGGACT; R: AGCAAGCCTCTCCTCCAAGTT	121
*dio2*	NM 212789.3	F: GCATAGGCAGTCGCTCATTT; R: TGTGGTCTCTCATCCAACCA	83
*ugt1ab*	NM 2213422.2	F: CCACCAAGTCTTTCCGTGTT; R: GCAGTCCTTCACAGGCTTTC	148
*ttr*	NM 001005598.2	F: CGGGTGGAGTTTGACACTTT; R: GCTCAGAAGGAGAGCCAGTA	110
*trα*	NM 131396.1	F:CTATGAACAGCACATCCGACAAGAG; R: CACACCACACACGGCTCATC	65
*trβ*	AF 302242.1	F: TGGGAGATGATACGGGTTGT; R: ATAGGTGCCGATCCAATGTC	90

### The extraction and analysis of thyroid hormone

The method for extraction of whole-body thyroid hormone was modified from Yu *et al* [32]. Briefly, 200 zebrafish larvae were homogenized in 0.3 mL ELISA buffer using a homogenizer. Then samples were disrupted by intermittent sonic oscillation for 15 min on ice. Next, the samples were centrifuged at 5000 × g for 10 min at 4°C. The supernatants were collected and stored at -80°C for T3 and T4 assay by using enzyme-linked immunosorbent assay (ELISA) kit, which was purchased from Uscnlife (Wuhan, China). The detection limits of T3 and T4 for this kit were 45.5 pg/mL and 1.5 ng/mL, respectively.

### Statistical analysis

The normality and homogeneity of variances were analyzed by Kolmogorov-Smirnov test and Leven’s test, respectively. Logarithmic transformation was performed for homogeneity of variance, if the data failed the Kolmogorov-Smirnov test. One-way analysis of variance (ANOVA) was applied to calculate the differences between the control and exposure groups followed by Dunnet test using SPSS 20.0 software (SPSS, Chicago, IL, USA). A value of *p*<0.05 was considered as statistically significant. All values were expressed as the means ± standard error (SEM).

## Results

### Developmental toxicity

Compared with the control groups, there were no significant developmental toxicity on zebrafish larvae at 168 hpf after exposure to DEHP (S2 Table). The parameters of hatching, growth, malformation and survival rate, body length and weight were shown in Table 2.

**Table 2:** Development index of zebrafish larvae after exposure to DEHP (0, 40, 100, 200, 400 μg/L) for 168 hpf^a^.

DEHP (μg/L)	0	40	100	200	400
Hatching (%)	92.67±1.76	90.00±1.33	89.78±1.35	92.22±2.19	91.11±0.44
Survival (%)	91.33±2.40	86.00±1.33	84.22±2.32	89.56±1.94	90.00±0.77
Malformation (%)	0.32±0.32	1.70±0.70	1.40±0.41	0.67±0.67	1.31±0.32
Weight (mg)	0.36±0.01	0.35±0.01	0.32±0.02	0.37±0.01	0.35±0.01
Length (mm)	3.29±0.02	3.41±0.07	2.99±0.05	2.99±0.13	3.05±0.16

^a^The values are the mean ± standard error (SEM) of six replicate groups.

### The assay of whole-body T4 and T3

**Fig 1:**
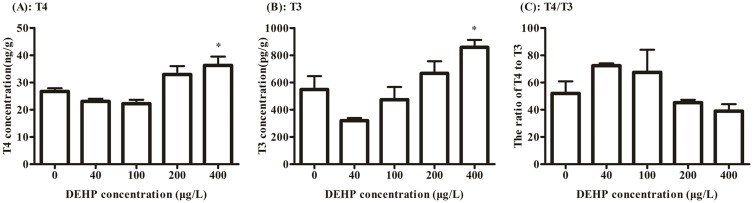
The whole-body levels of thyroid hormone T4 (A), T3 (B) and the T4/T3 (C) of zebrafish larvae at 168 hpf were determined after exposure to 0, 40, 100, 200, 400 μg/L of DEHP. The results were means ± SEM of three replicate samples. *P < 0.05 indicated significant difference between exposure groups and the control groups.

DEHP exposure increased the whole-body T4 and T3 contents in zebrafish larvae at the 400 μg/L concentration groups (*p* < 0.05) by 35.3% and 56.6%, respectively (Fig 1A and 1B). While the ratio of T4/T3 was dropped at the highest exposure groups without significant change, compared to the control (Fig 1C).

### mRNA expression profiles

**Fig 2:**
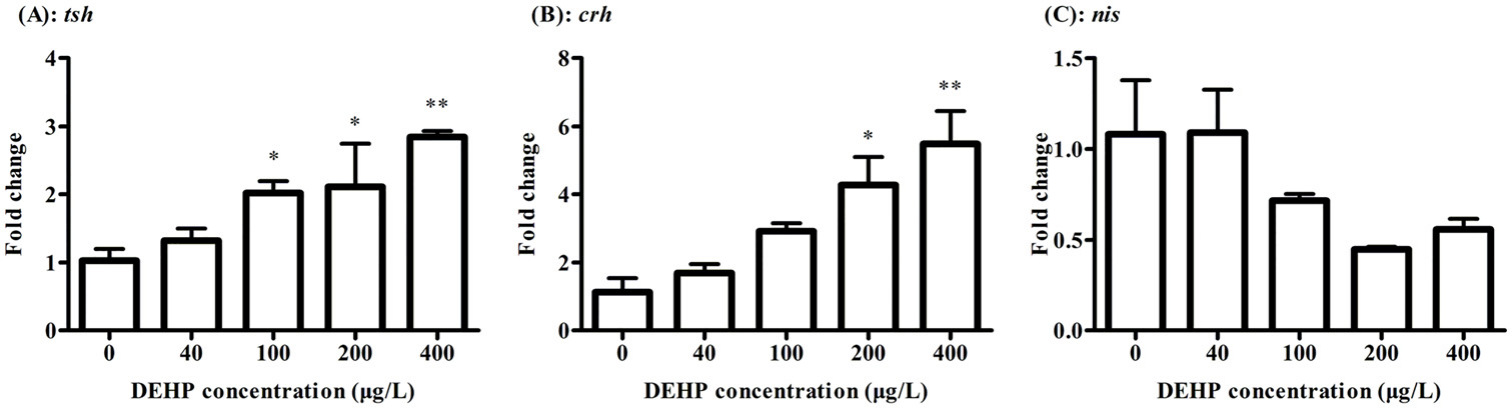
The expressions of *tshβ* (A) and *crh* (B) and *nis* (C) of zebrafish larvae were detected by real-time PCR after exposure to 0, 40, 100, 200, 400 μg/L of DEHP at 168 hpf. The results were means ± SEM of three replicate samples. Significant difference between exposure groups and the control groups was indicated by *P < 0.05 and **P < 0.01.

The gene expression of *tshβ* in zebrafish at 168 hpf stage was significantly up-regulated by 2.02, 2.11 and 2.84 folds, respectively, after exposure to 100, 200 and 400 μg/L of DEHP (Fig 2A). Compared to the control groups, the transcriptional level of the gene *crh* was significantly up-regulated by 4.27 and 5.48 folds, when exposed to 200 and 400 μg/L of DEHP, respectively (Fig 2B). The transcripts of *nis* were down-regulated, but had no significant change relative to the control (Fig 2C).

**Fig 3:**
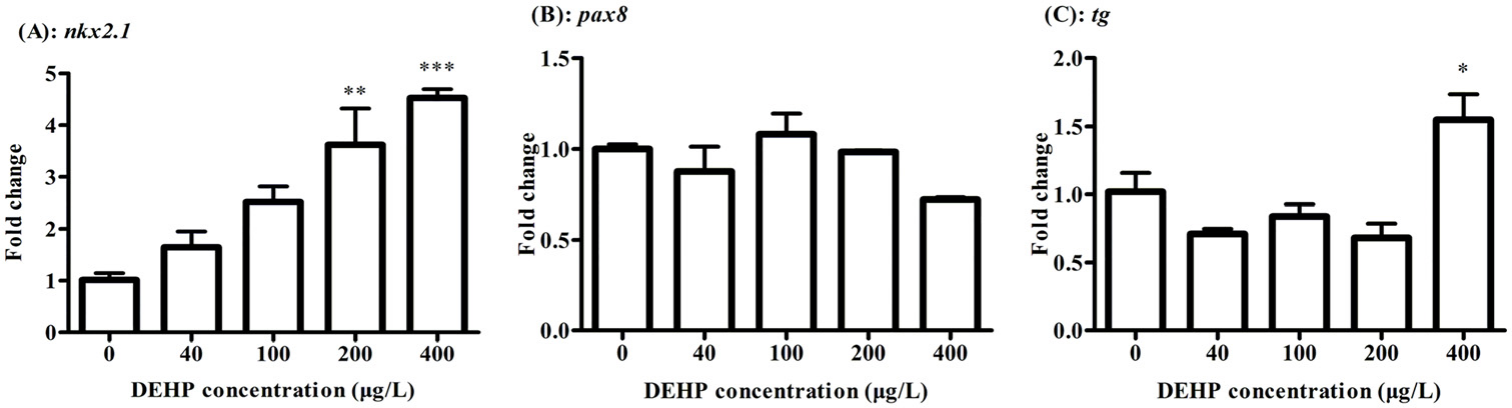
The expressions of *nkx2*.*1* (A) and *pax8* (B) and *tg* (C) were measured by real-time PCR after exposure to 0, 40, 100, 200, 400 μg/L of DEHP at 168 hpf in zebrafish larvae. The results were means ± SEM of three replicate samples. Significant difference between exposure groups and the control groups was indicated by *P < 0.05 and **P < 0.01 and ***P<0.001.

After exposure to 200 and 400 μg/L of DEHP, the transcriptional level of *nkx2*.*1* was significantly up-regulated by 3.62 and 4.52 folds compared to the control, respectively (Fig 3A). While *pax8* has no significant difference between the exposure and control groups (Fig 3B). In the 400 μg/L exposure groups, *tg* transcripts were significantly increased by 1.55 folds, but lower DEHP exposure caused no significant change (Fig 3C).

**Fig 4:**
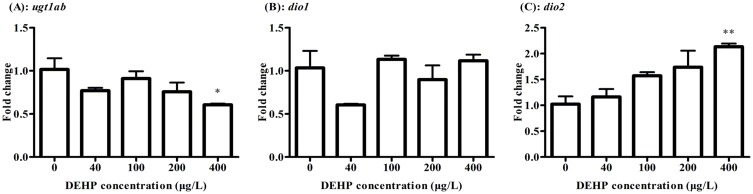
The expressions of *ugt1ab* (A) and *dio1* (B) and *dio2* (C) were checked by real-time PCR after exposure to 0, 40, 100, 200, 400 μg/L of DEHP at 168 hpf in zebrafish larvae. The results were means ± SEM of three replicate samples. Significant difference between exposure groups and the control groups was indicated by *P < 0.05 and **P < 0.01.

The mRNA level of *ugt1ab* involved in TH metabolism was significantly down-regulated by 1.68 folds after exposure to 400 μg/L of DEHP (Fig 4A). The two isoforms of deiodinases (Dio1 and Dio2) in zebrafish were checked. The expression of *dio1* had no significant differences (Fig 4B), while the *dio2* was increased significantly by 2.13 folds after exposure to 400 μg/L of DEHP (Fig 4C).

**Fig 5:**
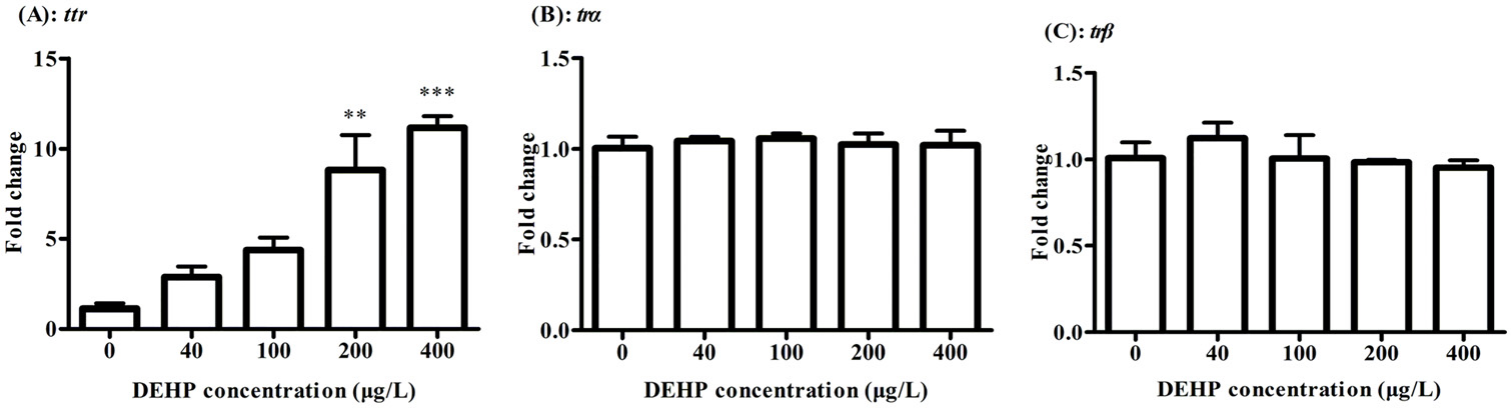
The expression change of *ttr* (A) and *trα* (B) and *trβ* (C) were measured by real-time PCR, after exposure to 0, 40, 100, 200, 400 μg/L of DEHP at 168 hpf in zebrafish larvae. The results were means ± SEM of three replicate samples. Significant difference between exposure groups and the control groups was indicated by *P < 0.05 and **P < 0.01 and ***P<0.001.

In this study, *ttr* transcripts were up-regulated significantly by 8.81 and 11.17 folds with a dose-dependent manner in 200 and 400 μg/L of DEHP exposure groups, respectively (Fig 5A). However, the expressions of thyroid hormone nuclear receptors isoforms, *trα* and *trβ* showed no significant change at 168 hpf stage in zebrafish after exposure to 0, 40, 100, 200, 400 μg/L of DEHP, respectively (Fig 5B and 5C).

## Discussion

The pollutants of PAEs family become the major global concerns because of their harmful effects on human in the surrounding environment. DEHP, the most common member of the class of phthalates, was widely used as plasticizers. DEHP has been reported to be a xenoestrogen to disrupt the organism’s endocrine stability. It is important to investigate the molecular toxicology of DEHP because of its high concentration found in the environment. In this study, we used zebrafish embryos and larvae as the model to evaluate the mechanism of thyroid endocrine disruption caused by DEHP. After exposure to DEHP, the whole-body thyroid hormones levels and the mRNA expression of key genes related to HPT axis were systematically investigated. The results showed that DEHP changed the THs levels and genes expression of HPT axis, suggesting the disturbance effects of DEHP in zebrafish embryos/larvae.

It has been reported that the pituitary gland secreted TSH to regulate the thyroid function, and the TSH secretion could be stimulated by CRH, which might function as a regulator of the HPT axis during larvae development in fish [26, 33, 34]. Thus, TSH assay can be used to detect the functional integrity of the pituitary and thyroid gland, and explain the mechanism of chemical disruption of thyroid function [35]. In this study, we found that the transcripts of *crh* and *tshβ* gene were significantly up-regulated after DEHP exposure, indicating that DEHP activated the HPT axis. Interestingly, in the highest exposure groups, the T4 level was also increased. A previous report on zebrafish larvae also showed that chemical exposure could up-regulate the transcription level of *tshβ* accompanying induction of T4 [36]. However, increasing expressions of *tshβ* and decreasing contents of T4 in zebrafish larvae were also reported in other studies, which was involved in negative feedback mechanism [23, 37]. Since TSH could initiate TH synthesis and release from the thyroid gland, we speculated that both up-regulation of *tsh* and *crh* triggered the increase of whole-body T4 level in the zebrafish larvae.

It was reported that Pax8 protein is required for the late differentiation of the follicular cells, and the transcription factor Nkx2.1 plays an essential role in thyroid development in fish [38]. Previous studies demonstrated that *nis* and *tg* are involved in TH synthesis, and *nkx2*.*1* is the main factor to regulate the expressions of *nis* and *tg* in the thyroid system [39, 40]. Here, we observed that exposure to DEHP significantly increased *nkx2*.*1* and *tg* transcription in the highest exposure groups. Zhai *et al*. also reported that the transcription of *nkx2*.*1* was significantly up-regulated by the increased expression of *tg* [23]. In our study, we speculated that the up-regulation levels of *nkx2*.*1* might promote the increased *tg* expression, which contributed to T4 induction.

As the major transport protein of THs, TTR plays an important role in the thyroid axis in fish [27]. Chemicals interfering with the TH binding ability to TTR may directly affect the concentration of free THs and its clearance rate in fish. The combined T4 would make it more stable to hepatic catabolism, resulting in a lesser clearance and an increased circulating TH [41, 42]. Moreover, previous studies showed that down-regulation of *ttr* gene expression was related to the reduction of T4 levels [23, 32]. In this study, the *ttr* mRNA level increased significantly after exposure to relatively higher concentrations of DEHP (200 μg/L and 400 μg/L), indicating that DEHP posed a potential risk to thyroid functions by affecting the binding and transporting ability of THs. In fish HPT axis, the biological activities of THs are primarily connected with genes binding to thyroid hormone receptors (TRs) [43]. Liu *et al*. reported that TRs acted as an inducible ligand-activated transcription factors and could affect the development of embryos [44]. In this study, exposure to DEHP did not alter TRs expression and also showed no significant effects on the development of zebrafish larvae, which was consistent with the previous reports [32, 45].

In fish, three types of deiodinases enzymes (Dio1, Dio2 and Dio3) play important roles in the regulation of TH levels. Studies proved that Dio1 had minimal effect on plasma TH homeostasis, but a major influence on iodine recovery and TH degradation. While Dio2 catalyzed the primary thyroxine (T4, a pro-hormone secreted from the thyroid follicles) and converted it to biological active triiodothyronine (T3) in the peripheral tissues [46, 47]. Previous study demonstrated that Dio2 played a critical role in T3 production at the early embryonic stages in zebrafish and increased the T3 availability [48]. It was also observed that the up-regulation of *dio2* was related to the increase of T3 contents, when zebrafish larvae were exposed to other chemicals [23, 49]. In this study, the expression of *dio2* was increased significantly after DEHP exposure at 400 μg/L, which may be partially responsible for the increase of the T3 content and the decline of T4/T3 ratio. The findings of increased *dio2* and T3 levels demonstrated that DEHP as the environment endocrine disrupting chemicals (EDCs) altered *dio2* expression and THs content via HPT axis in the zebrafish larvae.

It was reported that UGT (uridinedipgosphate glucoronosyltransferases) played important role in TH homeostasis by the T4 conjugation pathway [50]. Other studies proved that the decrease of T4 levels was accompanied with the increased *ugt1ab* expression after exposure to different pollutants [23, 32, 49]. In this study, it was observed that the gene expression of *ugt1ab* was down-regulated at 400 μg/L when exposed to DEHP. Thus, we supposed that the decrease of *ugt1ab* may be another reason for the increasing of T4 concentration in the zebrafish larvae.

THs were reported to be crucial for development, growth, differentiation and metabolism at early embryonic stages in zebrafish, especially for embryo to larval transitory phase and larval to juvenile transition phase [44, 51, 52]. Both lower concentrations of THs and excessive doses of THs might cause fish growth retardation [53, 54]. Fish larvae at early stages are more sensitive to toxic chemicals than adults because of the roles of thyroid hormones [55]. Hence, thyroid disruption can be used to reveal the toxicity of chemicals. In this study, the levels of whole-body T4 and T3 concentration were significantly up-regulated when exposed to two higher concentration of DEHP (200 μg/L and 400 μg/L), but there was no significant change for the developmental indexes, which was similar to a previous work on MEHP toxicity [23]. Reported studies indicated that 14 days or 15 days exposure to DE-71 or PFOS, respectively, in zebrafish larvae caused developmental toxicity [32, 45]. Thus, we speculate that a longer exposure to DEHP may cause the developmental toxicity in zebrafish larvae and more studies should be performed to check the chronic effects of DEHP on HPT axis.

In conclusion, this study showed that exposure to DEHP altered the whole-body THs levels and the expression of HPT axis related genes, proving the DEHP toxicity on thyroid endocrine in zebrafish larvae. This study revealed the possible mechanisms of DEHP disturbance on thyroid development. Due to DEHP as well as other PAEs is continually released into the natural environment, the potential risks caused by chronic exposure to PAEs on wild animals and human beings should be paid special attention.

## Supporting Information

S1 Fig Gene expression stability of the five candidate reference genes analyzed by the geNorm program. Pairwise variation analysis between the normalization factors NFn and NFn+1 was used to determine the optimal number of control genes for normalization.
(DOCX)

S1 Table The different concentrations of DEHP used in this study. (DOCX)

S2 Table Development index of zebrafish larvae after exposure to DEHP (0, 40, 100, 200, 400 ug/L) for 168 hpf^a^. (DOCX)

## References

[1] Lyche JL, Gutleb AC, Berqman A, Eriksen GS, Murk AJ, Ropstad E, et al Reproductive and developmental toxicity of phthalates. *J Toxicol Environ B Crit Rev*. 2009, 12(4):225–49. 10.1080/10937400903094091 20183522

[2] Rudel RA, Perovich LJ. Endocrine disrupting chemicals in indoor and outdoor air. *Atoms Environ*. 2009, 43(1):170–81. 10.1016/j.atmosenv.2008.09.025 PMC2677823 20047015

[3] Horn O, Nalli S, Cooper D, Nicell J. Plasticizer metabolites in the environment. *Water Res*. 2004, 38(17):3693–8. 15350420 10.1016/j.watres.2004.06.012

[4] Mathieu-Denoncourt J, Wallace SJ, de Solla SR, Langlois VS. Plasticizer endocrine disruption: Highlighting developmental and reproductive effects in mammals and non-mammalian aquatic species. *Gen Comp Endocrinol*. 2015, 219:74–88. 10.1016/j.ygcen.2014.11.003 25448254

[5] Oehlmann J, Oetken M, Schulte-Oehlmann U. A critical evaluation of the environment risk assessment for platicizers in the freshwater environment in Europe, with special emphasis on bisphenol A and endocrine disruption. *Environ Res*. 2008, 108(2):140–9. 18949832 10.1016/j.envres.2008.07.016

[6] Maqdouli S, Daqhrir R, Brar SK, Drogui P, Tyagi RD. Di 2-ethylhexylphthalates in the aquatic and terrestrial environment: a critical review. *J Environ Manage*. 2013, 127:36–49. 10.1016/j.jenvman.2013.04.013 23681404

[7] Huerta-Fontela M, Ventura F. Traceability of emerging contaminants from wastewater to drinking water. *Hdb Env Chem Vol*. 2008, 5:143–168.

[8] Wu P, Han G, Wang H. An investigation on phthalates in drinking water. *J Environ Health*. 1999, 16:338–9.[in Chinese].

[9] Zeng F, Cui K, Xie Z, Liu M, Li Y, Lin Y, et al Occurrence of phthalate esters in water and sediment of urban lakes in a subtropical city, Guangzhou, South China. *Environ Int*. 2008, 34(3):372–80. 17915327 10.1016/j.envint.2007.09.002

[10] Wang F, Xia X, Sha Y. Distribution of phthalic acid esters in Wuhan section of the Yangtze River, China. *J Harzard Mater*. 2008, 154(1–3):317–24. 10.1016/j.jhazmat.2007.10.028 18037235

[11] Erythropel HC, Maric M, Nicell JA, Leask RL, Yargeau V. Leaching of the plasticizer di(2-ethylhexyl)phthalate (DEHP) from plastic containers and the question of human exposure. *Appl Microbiol Biotechnol*. 2014, 98(24):9967–81. 10.1007/s00253-014-6183-8 25376446

[12] Chao KP, Huang CS, Wei CY. Health risk assessments of DEHP released from chemical protective gloves. *J Hazard Mater*. 2015, 283:53–9. 10.1016/j.jhazmat.2014.09.010 25261760

[13] Ghisari M, Bonefeld-Jorgensen EC. Effects of plasticizers and their mixture on estrogen receptor and thyroid hormone functions. *Toxicol Lett*. 2009, 189(1):67–77. 10.1016/j.toxlet.2009.05.004 19463926

[14] Weir SM, Wooten KJ, Smith PN, Salice CJ. Phthalate ester leachates in aquatic mesocosms: implications for ecotoxicity studies of endocrine disrupting compounds. *Chemosphere*. 2014, 103:44–50. 10.1016/j.chemosphere.2013.10.097 24309156

[15] Oehlmann J, Schulte-Oehlmann U, Kloas W, Jagnytsch O, Lutz I, Kusk KO, et al A critical analysis of the biological impacts of plasticizers on wildlife. *Philos Trans R Soc Lond B Biol Sci*. 2009, 364(1526):2047–62. 10.1098/rstb.2008.0242 19528055 PMC2873012

[16] Carnevali O, Tosti L, Speciale C, Peng C, Zhu Y, Maradonna F. DEHP impairs zebrafish reproduction by affecting critical factors in oogenesis. *PLoS One*. 2010, 5(4):e10201 10.1371/journal.pone.0010201 20419165 PMC2855362

[17] Corradetti B, Stronati A, Tosti L, Manicardi G, Carnevali O, Bizzaro D. Bis-(2-ethylexhyl) phthalate impairs spermatogenesis in zebrafish (Danio rerio). *Reprod Biol*. 2013, 13(3):195–202. 10.1016/j.repbio.2013.07.003 24011190

[18] Guo Y, Yang Y, Gao Y, Wang X, Zhou B. The impact of long term exposure to phthalic acid esters on reproduction in Chinese rare minnow (*Gobiocypris rarus*). *Environ Pollut*. 2015, 203:130–6. 10.1016/j.envpol.2015.04.005 25880617

[19] Matsumoto M, Hirata-Koizumi M, Ema M. Potential adverse effects of phthalic acid esters on human health: a review of recent studies on reproduction. *Requal Toxicol Pharmacol*. 2008, 50(1):37–49. 10.1016/j.yrtph.2007.09.004 17983696

[20] Ji Y, Wang F, Zhang L, Shan C, Bai Z, Sun Z, et al A comprehensive assessment of human exposure to phthalates from environment media and food in Tianjin, China. *J Hazard Mater*. 2014, 279:133–40. 10.1016/j.jhazmat.2014.06.055 25051237

[21] Chen X, Xu S, Tan T, Lee ST, Cheng SH, Lee FW, et al Toxicity and estrogenic endocrine disrupting activity of phthalates and their mixtures. *Int J Environ Res Public Health*. 2014, 11(3):3156–68. 10.3390/ijerph110303156 24637910 PMC3987027

[22] Ye T, Kang M, Huang Q, Fang C, Chen Y, Liu L. Accumulation of di(2-ethylhexyl) phthalate causes endocrine-disruptive effects in marine medaka (Oryzias melastigma) embryos. *Environ Toxicol*. 2014, 10.1002/tox.22028 25066029

[23] Zhai W, Huang Z, Chen L, Feng C, Li B, Li T. Thyroid endocrine disruption in zebrafish larvae after exposure to mono-(2-ethylhexyl) phthalate(MEHP). *PLoS One*. 2014, 9(3):e92465 10.1371/journal.pone.0092465 24658602 PMC3962405

[24] Blanton ML, Specker JL. The hypothalamic-pituitary-thyroid (HPT) axis in fish and its role in fish development and reproduction. *Crit Rev Toxicol*. 2007, 37(1–2):97–115. 17364706 10.1080/10408440601123529

[25] Fliers E, Kalsbeek A, Boelen A. Beyond the fixed setpoint of the hypothalamus-pituitary-thyroid axis. *Eur J Endocrinol*. 2014, 171(5):R197–208. 10.1530/EJE-14-0285 25005935

[26] De Groef B, Van der Geyten S, Darras VM, Kühn ER. Role of corticotrophin releasing hormones as a thyrotropin-releasing factor in non-mammalian vertebrates. *Gen Comp Endocrinol*. 2006, 146(1):62–68. 16337947 10.1016/j.ygcen.2005.10.014

[27] Power DM, Elias NP, Richardson SJ, Mendes J, Soares CM, Stantos CR. Evolution of the thyroid hormone-binding protein, transthyretin. *Gen Comp Endocrinol*. 2000, 119(3):241–55. 11017772 10.1006/gcen.2000.7520

[28] Sun HJ, Li HB, Xiang P, Zhang X, Ma LQ. Short-term exposure of arsenite disrupted thyroid endocrine system and altered gene transcription in the HPT axis in zebrafish. *Environ Pollut*. 2015, 205:145–52. 10.1016/j.envpol.2015.05.037 26057477

[29] Wang Q, Liang K, Liu J, Yang L, Guo Y, Liu C, et al Exposure of zebrafish embryos/larvae to TDCPP alters concentrations of thyroid hormones and transcription of genes involved in the hypothalamic-pituitary-thyroid axis. *Aquat Toxicol*. 2013, 126:207–13. 10.1016/j.aquatox.2012.11.009 23220413

[30] Dai YJ, Jia YF, Chen N, Bian WP, Li QK, Ma YB, et al Zebrafish as a model system to study toxicology. *Environ Toxicol Chem*. 2014, 33(1):11–7. 10.1002/etc.2406 24307630

[31] Segner H. Zebrafish (Danio rerio) as a model organism for investigating endocrine disruption. *Comp Biochem Physiol C Toxicol Pharmacol*. 2009, 149(2):187–95. 10.1016/j.cbpc.2008.10.099 18955160

[32] Yu L, Deng J, Shi X, Liu C, Yu K, Zhou B. Exposure to DE-71 alters thyroid hormone levels and gene transcription in the hypothalamic-pituitary-thyroid axis of zebrafish larvae. *Aquat Toxicol*. 2010, 97(3):226–33. 10.1016/j.aquatox.2009.10.022 19945756

[33] Manchado M, Infante C, Asensio E, Planas JV, Cañavate JP. Thyroid hormones down-regulate thyrotropin beta subunit and thyroglobulin during metamorphosis in the flatfish Senegalese sole (Solea senegalensis Kaup). *Gen Comp Endocrinol*. 2008, 155(2):447–55. 17888916 10.1016/j.ygcen.2007.07.011

[34] Chiamolera MI, Wondisford FE. Minireview: thyrotropin-releasing hormone and the thyroid hormone feedback mechanism. *Endocrinology*. 2009, 150(3):1091–6. 10.1210/en.2008-1795 19179434

[35] Zoeller RT, Tyl RW, Tan SW. Current and potential rodent screens and tests for thyroid toxicants. *Crit Rev Toxicol*. 2007, 37(1–2):55–95. 17364705 10.1080/10408440601123461

[36] Liu S, Chang J, Zhao Y, Zhu G. Changes of thyroid hormone levels and related gene expression in zebrafish on early life stage exposure to triadimefon. *Environ Toxcicol Pharmacol*. 2011, 32(3):472–7. 10.1016/j.etap.2011.09.002 22004968

[37] Yu L, Chen M, Liu Y, Gui W, Zhu G. Thyroid endocrine disruption in zebrafish larvae following exposure to hexaconazole and tebuconazole. *Aquat Toxicol*. 2013, 138–139:35–42. 10.1016/j.aquatox.2013.04.001 23685399

[38] Wendl T, Lun K, Mione M, Favor J, Brand M, Wilson SW, et al Pax2.1 is required for the development of thyroid follicles in zebrafish. *Development*. 2002, 129(15):3751–60. 12117823 10.1242/dev.129.15.3751

[39] Porazzi P, Calebiro D, Benato F, Tiso N, Persani L. Thyroid gland development and function in the zebrafish model. *Mol Cell Endocrinol*. 2009, 312(1–2):14–23. 10.1016/j.mce.2009.05.011 19481582

[40] Zoeller RT, Tan SW, Try RW. General background on the hypothalamic-pituitary-thyroid (HPT) axis. *Crit Rev Toxicol*. 2007, 37(1–2):11–53. 17364704 10.1080/10408440601123446

[41] Morgado I, Santos CR, Jacinto R, Power DM. Regulation of transthyretin by thyroid hormones in fish. *Gen Comp Endocrinol*. 2007, 152(2–3):189–97. 17289043 10.1016/j.ygcen.2006.12.017

[42] Fernie KJ, Shutt JL, Mayne G, Hoffman D, Letcher RJ, Drouillard KG, et al Exposure to polybrominated diphenyl ethers (PBDEs): changes in thyroid, vitamin A, glutathione homeostasis, and oxidative stress in American kestrels (Falco sparverius). *Toxicol Sci*. 2005, 88(2):375–83. 16120752 10.1093/toxsci/kfi295

[43] Marchand O, Safi R, Escriva H, Van Rompaey E, Prunet P, Laudet V. Molecular cloning and characterization of thyroid hormone receptors in teleost fish. *J Mol Endocrinol*. 2001, 26(1):51–65. 11174854 10.1677/jme.0.0260051

[44] Liu YW, Chan WK. Thyroid hormones are important for embryonic to larval transitory phase in zebrafish. *Differentiation*. 2002, 70(1):36–45. 11963654 10.1046/j.1432-0436.2002.700104.x

[45] Shi X, Liu C, Wu G, Zhou B. Waterborne exposure to PFOS causes disruption of the hypothalamus-pituitary-thyroid axis in zebrafish larvae. *Chemophere*. 2009, 77(7):1010–8. 10.1016/j.chemosphere.2009.07.074 19703701

[46] Orozco A, Valverde-R C. Thyroid hormone deiodination in fish. *Thyroid*. 2005, 15(8):799–813. 16131323 10.1089/thy.2005.15.799

[47] Van der Geyten S, Toquyeni A, Baroiller JF, Fauconneau B, Fostier A, Sanders JP, et al Hypothyroidism induces type I iodothyronine deiodinase expression in tilapia liver. *Gen Comp Endocrinol*, 2001, 124(3):333–42. 11742517 10.1006/gcen.2001.7722

[48] Walpita CN, Crawford AD, Janssens ED, Van der Geyten S, Darras VM. Type 2 iodothyronine deiodinase is essential for thyroid hormone-dependent embryonic development and pigmentation in zebrafish. *Endocrinology*. 2009, 150(1):530–9. 10.1210/en.2008-0457 18801906

[49] Chen Q, Yu L, Yang L, Zhou B. Bioconcentration and metabolism of decabromodiphenyl ether (BDE-209) result in thyroid endocrine disruption in zebrafish larvae. *Aquat Toxicol*. 2012, 110–111:141–8. 10.1016/j.aquatox.2012.01.008 22307006

[50] Hood A, Klaassen CD. Differential effects of microsomal enzyme inducers on in vitro thyroxine (T(4)) and triiodothyronine (T(3)) glucuronidation. *Toxicol Sci*. 2000, 55(1):78–84. 10788562 10.1093/toxsci/55.1.78

[51] Schnitzler JG, Klaren PH, Mariavelle E, Das K. The thyroid gland and thyroid hormones in sheepshead minnow (Cyprinodon variegatus) during early development and metamorphosis. *Fish Physiol Biochem*. 2016, 42(2):607–16. 10.1007/s10695-015-0163-5 26573854

[52] Power DM, Llewellyn L, Faustino M, Nowell MA, Björnsson BT, Einarsdottir IE, et al Thyroid hormones in growth and development of fish. *Comp Biochem Physiol C Toxicol Pharmacol*. 2001, 130(4):447–59. 11738632 10.1016/s1532-0456(01)00271-x

[53] Brown CL, Kim BG. Combined application of cortisol and triiodothyronine in the culture of larvae marine finfish. *Aquaculture*. 1995, 135:70–86.

[54] Lam TJ, Juario JV, Banno J. Effects of thyroxine on growth and development in post-yolksac larvae of milk-fish, Chanos chanos. *Aquaculture*. 1985, 46:179–84.

[55] Crane HM, Pickford DB, Hutchinson TH, Brown JA. Developmental changes of thyroid hormones in the fathead minnow, Pimephales promelas. *Gen Comp Endocrinol*. 2004, 139(1):55–60. 15474536 10.1016/j.ygcen.2004.07.004

