# Supplementary material for: The Effects of Disturbance on Hypothalamus-Pituitary-Thyroid (HPT) Axis in Zebrafish Larvae after Exposure to DEHP
Source: PLoS One. 2016 May 25;11(5):e0155762. doi: 10.1371/journal.pone.0155762 (PMC4880181; doi:10.1371/journal.pone.0155762)
Supplement: S1 Fig — Pairwise variation analysis between the normalization factors NFn and NFn+1 was used to determine the optimal number of control genes for normalization. (DOCX) [file pone.0155762.s001.docx]

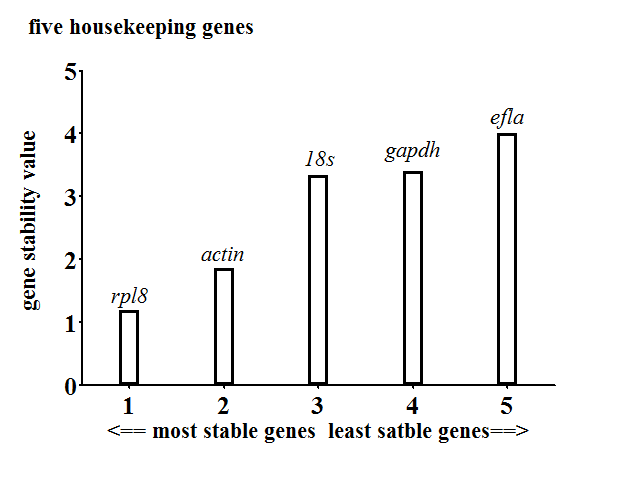


**S1 Fig. Gene expression stability of the five candidate reference genes analyzed by the geNorm program.** Pairwise variation analysis between the normalization factors NFn and NFn+1 was used to determine the optimal number of control genes for normalization.
