# Supplementary material for: The Effects of Disturbance on Hypothalamus-Pituitary-Thyroid (HPT) Axis in Zebrafish Larvae after Exposure to DEHP
Source: PLoS One. 2016 May 25;11(5):e0155762. doi: 10.1371/journal.pone.0155762 (PMC4880181; doi:10.1371/journal.pone.0155762)
Supplement: S1 Table — (DOCX) [file pone.0155762.s002.docx]

**S1 Table. The different concentrations of DEHP used in this study**

| DEHP buffers | 0 | 40 μg/L | 100 μg/L | 200 μg/L | 400 μg/L |
| --- | --- | --- | --- | --- | --- |
| Stock solution A (μL)**^a^** | - | - | - | 0.125 | 0.25 |
| Stock solution B (μL)**^a^** | - | 0.25 | 0.625 | - | - |
| DMSO (μL) | 1 | 0.75 | 0.375 | 0.875 | 0.75 |
| UltraPure water (mL) | 50 | 50 | 50 | 50 | 50 |

^a^The concentrations of DEHP stock solution (A: 8×10^7^ μg/L; B: 8×10^6^ μg/L)
