# Supplementary material for: The Effects of Disturbance on Hypothalamus-Pituitary-Thyroid (HPT) Axis in Zebrafish Larvae after Exposure to DEHP
Source: PLoS One. 2016 May 25;11(5):e0155762. doi: 10.1371/journal.pone.0155762 (PMC4880181; doi:10.1371/journal.pone.0155762)
Supplement: S2 Table — (DOCX) [file pone.0155762.s003.docx]

| **DEHP (ug/L)** | **0** | **40** | **100** | **200** | **400** |
| --- | --- | --- | --- | --- | --- |
| Hatching (%) | 92.67±1.76 | 90.00±1.33 | 89.78±1.35 | 92.22±2.19 | 91.11±0.44 |
| P value | - | 0.570 | 0.508 | 0.999 | 0.880 |
| Survival (%) | 91.33±2.40 | 86.00±1.33 | 84.22±2.32 | 89.56±1.94 | 90.00±0.77 |
| P value | - | 0.197 | 0.068 | 0.900 | 0.960 |
| Malformation (%) | 0.32±0.32 | 1.70±0.70 | 1.40±0.41 | 0.67±0.67 | 1.31±0.32 |
| P value | - | 0.233 | 0.412 | 0.963 | 0.484 |
| Weight (mg) | 0.36±0.01 | 0.35±0.01 | 0.32±0.02 | 0.37±0.01 | 0.35±0.01 |
| P value | - | 0.991 | 0.103 | 0.477 | 0.998 |
| Length (mm) | 3.29±0.02 | 3.41±0.07 | 2.99±0.05 | 2.99±0.13 | 3.05±0.16 |
| P value | - | 0.848 | 0.195 | 0.193 | 0.348 |

**S2 Table. Development index of zebrafish larvae after exposure to DEHP (0, 40, 100, 200, 400 ug/L) for 168 hpf^a^**

^a^The values are the mean ± standard error (SEM) of six replicate groups.
